# The CCN Family Proteins: Modulators of Bone Development and Novel Targets in Bone-Associated Tumors

**DOI:** 10.1155/2014/437096

**Published:** 2014-01-14

**Authors:** Po-Chun Chen, Hsu-Chen Cheng, Shun-Fa Yang, Chiao-Wen Lin, Chih-Hsin Tang

**Affiliations:** ^1^Graduate Institute of Basic Medical Science, China Medical University, Taichung 40402, Taiwan; ^2^Department of Medical Research, Chung Shan Medical University Hospital, Chung Shan Medical University, Taichung 40201, Taiwan; ^3^Department of Life Sciences, National Chung Hsing University, Taichung 40227, Taiwan; ^4^Institute of Medicine, Chung Shan Medical University, Taichung 40201, Taiwan; ^5^Institute of Oral Sciences, Chung Shan Medical University, Taichung 40201, Taiwan; ^6^Department of Dentistry, Chung Shan Medical University Hospital, Taichung 40201, Taiwan; ^7^Department of Pharmacology, School of Medicine, China Medical University, Taichung 40402, Taiwan; ^8^Department of Biotechnology, College of Health Science, Asia University, Taichung 41354, Taiwan

## Abstract

The CCN family of proteins is composed of six extracellular matrix-associated proteins that play crucial roles in skeletal development, wound healing, fibrosis, and cancer. Members of the CCN family share four conserved cysteine-rich modular domains that trigger signal transduction in cell adhesion, migration, proliferation, differentiation, and survival through direct binding to specific integrin receptors and heparan sulfate proteoglycans. In the present review, we discuss the roles of the CCN family proteins in regulating resident cells of the bone microenvironment. In vertebrate development, the CCN family plays a critical role in osteo/chondrogenesis and vasculo/angiogenesis. These effects are regulated through signaling via integrins, bone morphogenetic protein, vascular endothelial growth factor, Wnt, and Notch via direct binding to CCN family proteins. Due to the important roles of CCN family proteins in skeletal development, abnormal expression of CCN proteins is related to the tumorigenesis of primary bone tumors such as osteosarcoma, Ewing sarcoma, and chondrosarcoma. Additionally, emerging studies have suggested that CCN proteins may affect progression of secondary metastatic bone tumors by moderating the bone microenvironment. CCN proteins could therefore serve as potential therapeutic targets for drug development against primary and metastatic bone tumors.

## 1. Introduction

The extracellular matrix (ECM) primarily serves as a scaffold for the organization of cells into tissues. However, it has also been recognized as a multifunctional modulator of cellular behavior [1, 2]. Through direct interaction, ECM proteins could modulate activities of many growth factors, cytokines, chemokines, and extracellular proteins or elicit signal transduction cascades, thus regulating diverse cellular functions. Recently, many studies have focused on a group of matrix proteins known as “matricellular” proteins for their function in extracellular signal modulation and coordination [3]. The CCN family, a small group of such matricellular proteins, is composed of six structurally conserved secreted proteins that have been identified in several biological studies [4–6].

The CCN family is named after its three initially discovered members: cysteine rich 61 (Cyr61, CCN1), connective tissue growth factor (CTGF, CCN2), and nephroblastoma overexpressed (Nov, CCN3) [7]. The CCN family includes three other members, Wnt induced secreted proteins 1–3 also known as CCN4, CCN5, and CCN6. The CCN members share approximately 40% to 60% amino acid homology and comprise a signal peptide followed by 4 functional domains with 38 conserved cysteine residues [8]. In general, the common structure consists of an N-terminal signal peptide followed by an insulin-like growth factor binding protein domain (IGFBP), a von Willebrand type C repeat (VWC), a thrombospondin type I domain (TSP-1), and a cysteine knot carboxyl terminal (CT) [9]. The CCN proteins regulate cell adhesion, migration, proliferation, and differentiation to modulate variant biological functions including tumorigenesis, chondrogenesis, osteogenesis, angiogenesis, apoptosis, and hematopoiesis [5]. Numerous studies have shown that the biological functions of CCN proteins are mediated through interactions with cell surface receptors such as integrins, heparan sulfate proteoglycans (HSPGs), Notch1, neurotrophic tyrosine kinase receptor type 1 (TrkA), and low-density lipoprotein receptor-related proteins (LRPs). Moreover, CCN proteins could interact with other components outside of the cells such as ECM proteins, including fibronectin and fibulin 1C, and growth factors, including bone morphogenetic proteins (BMPs), tumor growth factor beta (TGF-*β*), and vascular endothelial growth factor [4].

The CCN family proteins were initially classified as growth factors. However, later studies showed that CCN proteins are matricellular proteins that modify cellular responses to extracellular factors via direct binding to cell surface receptors [5, 10–14]. Importantly, *in vivo* studies have indicated that aberrant expression of CCN proteins is involved in many diseases, including arthritis, atherosclerosis, fibrosis, diabetic nephropathy, retinopathy, and cancer [15]. Although the CCN proteins were discovered a decade ago, their mechanisms of action remain ambiguous. In the present report, we summarize recent literature that focuses on the regulation and function of CCN proteins in various bone tumors, discuss their potential as diagnostic markers and therapeutic targets, and review the recent therapeutic strategies targeting these proteins.

## 2. Receptors of CCN Family Proteins

CCN proteins were shown in previous studies to exert their function through direct binding to integrins or HSPGs. The interaction between CCN proteins and integrins was first discovered in 1998 by Kireeva et al. [16]. To date, at least 8 integrins have been demonstrated to interact with CCN proteins [4], which, however, do not possess the typical integrin binding sequence “RGD.” Therefore, the interaction is thought to occur through nontypical binding sites, which is confirmed by site-directed mutagenesis that inhibits the biological activities induced by integrin binding. For example, a GVCTDGR sequence in CT domain of CCN2 interacts with integrin *α*5*β*1 binding site and regulates CCN2-stimulated functions [17, 18]. In addition, CCN3 has been shown to bind to Notch and regulate myoblast and osteoblast functions [19, 20].

Other coreceptors are also involved in CCN protein signaling regulation. Cell surface HSPGs such as syndecan-4, perlecan, decorin, and biglycan have been reported to regulate CCN protein function in human fibroblasts [21–25]. CCN2 could also bind to coreceptors of the Wnt receptor LDL-receptor related protein 6 (LRP6) and LRP1 through variant modules [26, 27]. Moreover, Edwards et al. reported that CCN2 binds to TrkA (also known as neurotrophic tyrosine kinase receptor type 1) (NTRK1) in human mesangial and glioma cells [28, 29] and that TrkA serves as a co-receptor with integrins in this interaction. In summary, the complexity of receptors and coreceptors contributes to the unique activities and functions of CCN proteins in various cell types.

## 3. Functions of CCN Family Proteins

### 3.1. Adhesion and Migration

As expected from matricellular proteins modulating ECM signaling, the most familiar functions of CCN proteins are their roles in cell adhesion and migration. For example, CCN1 and CCN2 regulate adhesion in several types of cells [30]. In human skin fibroblasts, CCN1- and CCN2-regulated cell attachment is mediated through integrin *α*6*β*1 and HSPGs [31]. In vascular smooth muscle cells, endothelial cells, and fibroblasts, CCN3 promotes cell adhesion through integrins and HSPGs [5]. Despite the lack of the RGD motif, the canonical binding motif for integrins, CCN3 could interact with many integrin receptors such as integrin *α*6*β*1, *α*5*β*1, *α*v*β*3, *α*v*β*5, *α*2*β*1, *α*3*β*1, and *α*7*β*1. CCN proteins can mediate cell migration through interaction with cell surface receptors. Previous studies have shown that all CCN proteins could regulate cell migration in many cell types. CCN1, CCN2, and CCN3 proteins promote cell migration in different types of cells [4]. CCN4 is involved in the migration and proliferation of vascular smooth muscle cells [32]. CCN5, however, is an important negative regulator of motility through matrix metalloproteinase (MMP)-2 gene expression modulation [33]. Finally, CCN6 stimulates migration of undifferentiated mesenchymal stroma cells [34].

### 3.2. Cell Survival and Apoptosis

Adhesion to the ECM is a crucial process to promote cell survival, whereas detachment from the ECM induces rapid cell death. The mechanism of CCN proteins regulating cell fates varies in different cell types. For example, CCN1 can promote cell survival in human umbilical vein endothelial cells through integrin *α*v*β*3 [35] but induces fibroblast apoptosis through integrin *α*6*β*1-HSPG syndecan-4 interaction [24]. These results suggest that specific CCN matricellular protein can either induce or suppress apoptosis via variant receptor interaction in a cell type-specific manner.

### 3.3. Proliferation

The first discovered CCN protein CCN1, also known as Cyr61, was believed to be a classic growth factor. However, later efforts established that CCN1, instead of being a growth factor itself, enhanced the activity of some growth factors such as fibroblast growth factor and platelet-derived growth factor [36]. CCN2 has been demonstrated to induce proliferation of chondrocytes through the MAPK/ERK signaling pathway [37], and knockdown of CCN2 expression inhibits cell proliferation and increases apoptosis [38]. CCN3, however, has been reported to have negative regulatory properties, and its abnormal expression is associated with cancer progression [39]. Other studies have otherwise indicated that high CCN3 expression is associated with increased proliferation rates or tumor promoting potential in many cancer types [40]. The ambiguous effects of CCN3 therefore require further investigation.

### 3.4. Angiogenesis

CCN proteins have been suggested as potent angiogenic modulators, with activity mediated by interactions with different integrins and growth factors [41]. Treatments with recombinant CCN1, CCN2, and CCN3 increase angiogenesis *in vivo*, demonstrated via subcutaneous injection into corneas and the chick chorioallantoic membrane assay [5]. CCN1 and CCN2 play crucial roles in embryonic angiogenesis. Knockdown of CCN1 expression induces cardiovascular defects and is associated with embryonic lethality due to placental vascular inefficiency and compromised blood vessels [42]. CCN2, however, regulates angiogenesis via a different developmental process. *In vivo *results show that CCN2-null mutant mice show angiogenic deficiency in the growth plates during endochondral bone formation [43]. In addition, CCN3 has been demonstrated as a novel angiogenic regulator acting directly on endothelial cells to stimulate proangiogenic activities and as an angiogenesis inducer *in vivo *[44]. However, the roles of CCN4, CCN5, and CCN6 in angiogenesis remain poorly understood.

### 3.5. Inflammation

Abundant evidences indicate that CCN proteins are involved in inflammatory responses [45]. CCN protein expression is tightly regulated by different inflammatory mediators, including cytokines such as tumor necrosis factor alpha (TNF-*α*), interleukin (IL)-1*β*, and TGF-*β*, or by small factors such as prostaglandins, nitric oxide, histamine, and serotonin. Moreover, viral or bacterial infection also induces CCN proteins expression. Recognized as being encoded by an immediate early gene induced by environmental changes, CCN proteins subsequently regulate activity and expression of inflammatory cytokines and chemokines. For example, a recently published study showed that CCN1 promoted a proinflammatory program in murine macrophages. Bai et al. reported that CCN1 induced the expression of proinflammatory cytokines such as TNF-*α*, IL1-*α*, IL1-*β*, and IL-6; chemokines; and regulators of oxidative stress and inhibited the expression of anti-inflammatory factors such as TGF-*β* [46]. In addition, numerous studies have demonstrated a pivotal role of CCN proteins in chronic inflammatory diseases such as atherosclerosis, rheumatoid arthritis, inflammatory kidney diseases, and Alzheimer disease [45]. Therefore, CCN proteins may be classified as a new class of inflammatory regulators.

## 4. The Role of CCN Proteins in Bone

Bone is a complex tissue composed of two major cell types, bone resorption osteoclasts and bone formation osteoblasts, responsible for bone remodeling. Another cell population resident in the cartilage is chondrocytes. Abundant evidence suggests that CCN proteins regulate the differentiation of these cells (osteoblasts, osteoclast, and chondrocyte) [47]. In addition, CCN proteins are highly regulated during chondrogenic and osteogenic differentiation in mesenchymal stem cells [48–50]. However, CCN proteins can play either a positive or a negative regulatory role in skeletogenesis, which has been demonstrated both* in vitro* and* in vivo *[5, 51].

CCN1 has been detected in mouse limb bud mesenchymal cells during chondrogenesis and has been shown to promote chondrogenic differentiation through expression of type II collagen [52]. In another study, the tightly regulated CCN1 expression was shown to be involved in Wnt3A-induced osteoblast differentiation of mesenchymal stem cells [50]. Moreover, CCN1 has been shown to promote osteogenesis by increasing osteoblast differentiation while inhibiting osteoclast formation [53].

CCN2 is the most discussed member of the CCN protein family, accounting for approximately 50% of all reports published on the subject [54], most of which focus on the role of CCN2 in fibrosis and osteo/chondrogenesis. These reports indicate that CCN2 plays a crucial role in embryogenesis and skeletogenesis. For example, CCN2 has been shown to promote proliferation, chondrogenic differentiation, and chondrocyte maturation [55, 56]. CCN2 expression is high in the vascular tissue and maturing chondrocytes of the embryo and is important for cell proliferation and matrix remodeling during chondrogenesis [43]. In addition, CCN2 could also interact with many BMPs, important bone formation regulators, to regulate chondrocyte proliferation and differentiation [57, 58]. Finally,* in vivo* results have shown that CCN2 deficiency leads to skeletal dysmorphisms caused by impaired chondrocyte proliferation and reduced ECM composition in the growth plate [43].

Reported results on CCN3, however, are contradicting. CCN3 has been found to inhibit osteoblastogenesis and cause osteopenia, through antagonizing BMP-2, and Wnt activity in mice [59]. CCN3 has also been shown to inhibit osteoblast differentiation by neutralizing BMP2, a well-known enhancer of osteoblastogenesis in MC3T3 osteoblast precursor cells, in *in vivo *studies [20]. In another study, CCN3 showed antagonistic properties, inhibiting osteoblastogenesis and osteoblastic function through BMP2 neutralization and impairment of Wnt3 signaling [60]. In summary, these results suggest CCN3 as a negative regulator of osteoblastogenesis through multiple mechanisms including BMP2 and Wnt signaling or via activation of the Notch1 pathway. In contrast, a recent study has indicated that CCN3 promotes osteoblast differentiation and bone mineralization by upregulation of BMP-4, a well-known inducer of osteoblast differentiation [61]. In that study, a lower dose CCN3 (30 ng/mL) increased osteoblast differentiation whereas a higher dose (600 ng/mL) exhibited an opposite phenomenon, suggesting concentration-dependent mechanisms of action for CCN3.

Studies on the other three members of the CCN family are scarce, except for a report on CCN6. In that study, point mutations in CCN6 were shown to relate to the autosomal recessive skeletal disease progressive pseudorheumatoid dysplasia, a human disease resulting in progressive degeneration of articular cartilage [54].

## 5. The Role of CCN Family Proteins in Primary Bone Cancers

CCN proteins are tightly regulated in osteo/chondrogenic cell lineages and are involved in skeletogenesis. Abnormal levels or altered forms of CCN proteins are associated with tumor progression. We hereby discuss the correlation between CCN proteins and primary bone cancers (Table 1).

### 5.1. Osteosarcoma

Osteosarcoma is the most common primary bone tumor found in children and young adults. The existing literature suggests that osteosarcoma might originate from mesenchymal cells with osteoblastic features [73–75]. The CCN1 expression level in osteosarcoma biopsies has been shown to correlate with poor prognosis, regardless of metastatic or nonmetastatic disease. Moreover, an *in vivo* murine model showed that overexpression of CCN1 in the low-metastatic human SaOS-2 osteosarcoma cell line increased cell proliferation and promoted lung metastasis [62]. Fromigue et al. also demonstrated that CCN1 protein expression was higher in human osteosarcoma than in normal bone tissue and was most highly expressed in metastatic tissues. They also found that CCN1 knockdown inhibited *in vitro* osteosarcoma cell invasion and migration as well as *in vivo* lung metastases in mice [63]. Therefore, these results demonstrate great potential for CCN1 as a novel prognosis marker and therapeutic target in osteosarcoma. In addition, another study showed that CCN3 was expressed in primary tumors of osteosarcoma patients and that a high CCN3 expression level was associated with an increased risk of developing lung metastases [64]. CCN4 also showed similar correlation like CCN1 and CCN3 in osteosarcoma. In our previous work, we show that the expression of CCN4 in osteosarcoma patients was significantly higher than that in normal bone and corrected with tumor stage. CCN4 increases cell motility through upregulating matrix metalloproteinase (MMP)-2 and MMP-9 expression [65].

### 5.2. Ewing Sarcoma

Ewing sarcoma is the second most common malignant bone tumor that mainly occurs in children. CCN3 is expressed in approximately 30% of all Ewing sarcoma cases, and its expression is associated with a lower survival rate [64]. In a study by Benini et al., overexpression of CCN3 led to decreased *in vitro* cell proliferation and soft-agar growth in Ewing sarcoma cells and *in vivo *tumorigenicity in nude mice. However, these Ewing sarcoma cells showed increased migration and invasion in Matrigel [67]. Finally, an immunohistochemistry study on 170 human Ewing sarcoma specimens showed that the expression of CCN3 was higher in recurrences and metastases than in primary tumors. The same study also suggested that a low level of CCN3 expression was associated with better patient prognosis [66].

### 5.3. Chondrosarcoma

Chondrosarcoma is the second most common malignancy of the bone, associated with a poor response to currently used chemotherapy and radiation treatment, making chondrosarcoma management a complicated challenge [76, 77]. All CCN proteins have been demonstrated to be involved in chondrosarcoma progression and malignancy except for CCN5. The CCN proteins have been shown to promote cell migration through upregulation of various genes such as MMP-2, MMP-13, and intercellular adhesion molecule 1 (ICAM-1) [68–72]. These results suggest that CCN proteins might regulate common biological functions in chondrosarcoma. Other cellular functions regulated by CCN proteins such as adhesion, proliferation, survival, apoptosis, and angiogenesis may also be involved in CCN protein-regulated tumorigenesis. Interestingly, CCN proteins promote expression of MMPs, important regulators of ECM, which might explain the prometastatic effects exerted by the CCN family. The tumor microenvironment could also significantly influence chondrosarcoma malignancies. A previous study indicated that the tumor microenvironment could affect CCN2 gene expression in Swarm rat chondrosarcoma tumors, suggesting that CCN2 may play a role in chondrosarcoma development and progression [78].

## 6. The Role of CCN Family Proteins in Metastatic Bone Cancers

Bone metastasis is a common complication of advanced cancer, occurring when cancer cells from the primary tumor spread to the bone. Prostate, breast, and lung cancers are most likely to result in bone metastasis. As CCN proteins have important roles in the differentiation and function of bone resident cells, they have been implicated in the progression of bone metastases from other cancers (Table 2).

### 6.1. Breast Cancer Metastasis to the Bone

Breast cancer shows a high predilection for metastasis to the bone, causing bone pain, pathological fractures, hypercalcemia, spinal cord compression, and immobility [93]. In a cohort of 122 human breast tumors and 32 normal breast specimens, significantly elevated levels of CCN1 were shown to be associated with poor prognosis, nodal involvement, and metastatic disease [79]. CCN1 is a potent proangiogenic molecule, and a previously published study suggested the critical role of CCN1 in the Hedgehog-influenced proangiogenic tumor microenvironment [94]. CCN1 has also been recommended as a candidate target for breast cancer bone metastases. Espinoza et al. found that zoledronic acid, a bisphosphonate currently used to treat breast cancer bone metastases, downregulated CCN1, thus inhibiting tumor growth [80]. Moreover, the anti-human CCN1 antibody, denoted as 093G9, was shown to inhibit breast cancer cell migration and invasion through upregulation of the MMP inhibitors TIMP1 and TIMP2. *In vivo* mouse model results showed that 093G9 also inhibited primary tumor growth and spontaneous lymph node metastases [81].

CCN2 was found to be overexpressed in tumor cells from human bone metastases compared to a normal human epithelial cell line [82]. In addition, another report indicated that CCN2 was significantly overexpressed in metastatic tumor cells compared to disseminated tumor cells [83], further supporting the previously mentioned evidence. An *in vivo* mouse model study was performed to investigate the role of CCN2 in osteolytic metastasis by breast cancer cells. The results showed that CCN2 was crucial for osteolytic metastasis and was induced by protein kinase A- and protein kinase C-dependent activation of ERK1/2 signaling by parathyroid hormone-related protein (PTHrP). The authors also found that osteolytic metastasis accompanied by the PTHrP-CCN2 signaling pathway was efficiently abolished by a CCN2 neutralizing antibody [84]. Another previously published study investigated the mechanism of osteolytic bone metastasis by selecting human breast cancer cell line subpopulations with elevated metastatic activity and found that IL-11 and CCN2 expressions were further increased by the prometastatic cytokine TGF*β*. These results elucidated a mechanism for the prometastatic activity of these cytokines in the bone [85].

However, studies on CCN3 have reported contradicting results. In a different cohort of 122 human breast tumors and 32 normal breast specimens, the expression of CCN3 was found to be lower in tumor tissues when compared to normal specimens [79]. Interestingly, in another study, a microarray profile derived from 58 breast cancer metastases showed CCN3 to be highly expressed in bone metastases when compared to other metastases (lung, brain, and liver) [86]. Moreover, Véronique et al. found that CCN3 was highly expressed in bone metastasis samples from breast cancer patients. They also demonstrated that CCN3 increased the bone metastatic potential of 66cl4 cells, which are breast cancer cells metastasizing to the lungs [87].

The balance between bone formation and resorption is a significant factor in the development of bone metastasis. In accordance with this opinion, CCN3 was shown to impair osteoblast differentiation and affect receptor activator of NF-*κ*B ligand (RANKL)/osteoprotegerin ratios of osteoblasts, thereby enhancing osteoclastogenesis. CCN3 was also shown to promote osteoclast differentiation through a RANKL-dependent pathway, which involves calcium oscillations and nuclear factor of activated T-cell nuclear translocation [87].

In contrast, studies on CCN6 indicated that it inhibited breast cancer metastasis. In clinical specimens, CCN6 expression was shown to inversely correlate with invasive breast carcinomas. Moreover, CCN6 was shown to inhibit invasion and metastasis of breast cancer *in vivo*. The mechanism of CCN6-inhibited breast cancer progression was shown to be mediated by the BMP4/TAK1/p38 pathway, which could induce epithelial-mesenchymal transition, cell invasion, and metastasis [88]. Decline of CCN6 protein expression was demonstrated to sufficiently activate the phosphatidylinositol 3-kinase/Akt signaling pathway, thus promoting growth factor-independent survival that is triggered by resistance to detachment-induced cell death (anoikis) [95]. The role of CCN6 in breast cancer metastasis has been proved. However, the role of CCN6 in bone metastatic breast cancer remains poorly understood and further studies are necessary.

### 6.2. Prostate Cancer Metastasis to the Bone

Prostate cancer is the most commonly diagnosed malignancy in the United States and other Western countries, and bone metastasis is a common complication associated with advanced prostate cancer [96–98]. Prostate cancer bone metastases are most often characterized as osteoblastic lesions as opposed to osteolytic lesions with decreased bone mineral density. Increasing evidence suggests that prostate cancer cells synchronize the combined osteoclastic and osteoblastic activity occurring in the bone microenvironment [99–101]. However, the role of CCN proteins in prostate cancer metastasis to the bone is discussed relatively scarcely.

CCN1 has been implicated in tumorigenesis and metastasis of prostate cancer cells [89]. CCN1 activates Ras-related C3 botulinum toxin substrate 1 and its downstream targets, including phosphorylated c-Jun N-terminal kinase, E-cadherin, and p27 (kip1), key molecules involved in cell growth, migration, and invasion. *In vivo* mouse model results revealed that CCN1 increased the metastatic potential of prostate cancer cells. The correlation of CCN1 and prostate cancer bone metastases, however, needs to be confirmed.

CCN3 has been demonstrated to have prometastatic potential in prostate cancer in our previous study [90]. We found that CCN3 increased cell migration through the upregulation of ICAM-1 expression. Knockdown of CCN3 expression markedly inhibited cell migration *in vitro* and tumor growth in bone and bone metastasis *in vivo*. Moreover, our latest study revealed the critical role of CCN3 in prostate cancer bone metastases [91]. An immunohistochemistry study on normal prostate tissues, primary tumors, and bone metastasis samples obtained from patients revealed that CCN3 expression levels were higher in patients with bone metastasis and positively correlated with malignancy in human prostate cancer cells. In agreement with the study by Véronique et al. [87], our results showed that the prostate cancer-secreted CCN3 induced osteoclastogenesis through a RANKL-dependent pathway. Moreover, the focal adhesion kinase/Akt/p38/NF-*κ*B signaling pathway was found to be involved in CCN3-mediated receptor activator of NF-*κ*B expression and RANKL-dependent osteoclastogenesis. Experiments with intratibia injection of prostate cancer cells also proved that CCN3 enhanced osteoclast activity and bone metastasis* in vivo.*

CCN4 has been shown to play similar roles to CCN3 in prostate cancer [92]. An increased expression level of CCN4 has been found in prostate cancer tissue in the early stages, sera of patients, and carcinoma tissues of the mouse prostate cancer model TRAMP, which spontaneously develops to prostate carcinomas. Injections of CCN4 neutralizing antibodies were shown to reduce local tumor growth in a mouse xenograft model. These results suggest that CCN4 expression plays significant roles in both tumor growth and its metastasis to bone.

## 7. Perspectives

The CCN family proteins are multifunctional cytokines that regulate signals from the ECM. They are involved in many cellular processes and exert their functions through modulating various components including ECM proteins, transmembrane proteins, growth factors, and cytokines in the cell microenvironment. As they have crucial roles in osteo/chondrogenesis during development, abnormal expression of CCN proteins is implicated in tumors that grow in the bone microenvironment such as primary bone tumors and bone metastases. However, the roles of CCN proteins in osteo/chondrogenesis in development vary. The complicated interaction of CCN family proteins and other components outside the cells may contribute to unique activities and functions of CCN proteins. Emerging results suggest that the ultimate outcome of cellular responses modulated by CCN family proteins may also depend on the level of CCN expression. Although the available results suggest ambiguous roles of the CCN family proteins, they reveal the significance of CCN proteins in the regulation of bone homeostasis and turnover.

**Figure 1:**
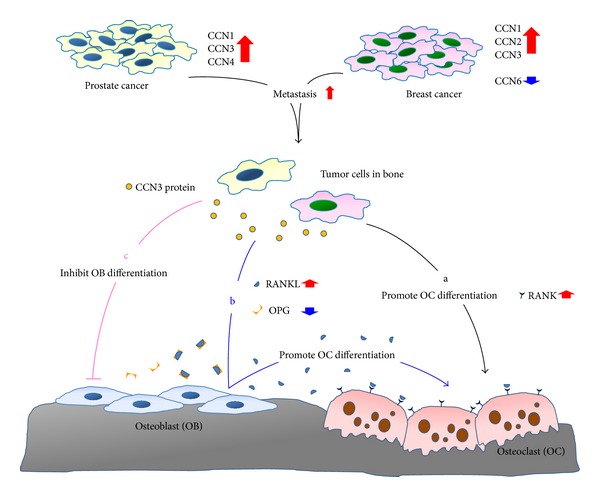
CCN family proteins involved in tumor metastasis and the mechanism of CCN3-modulated osteolytic bone metastasis. Tumors secrete different CCN proteins in prostate cancer (such as CCN1, CCN3, and CCN4) or breast cancer (such as CCN1, CCN2, CCN3, and CCN6) that regulate tumor metastasis. When tumor cells metastasize to the bone microenvironment, the secreted CCN protein (such as CCN3) promotes osteolytic bone metastasis in bone microenvironment through 3 different mechanisms. (a) CCN3 directly enhances osteoclasts formation through upregulating RANK expression, the crucial regulator of osteoclastogenesis. (b) CCN3 indirectly enhances osteoclast formation through affecting the RANKL/OPG secretion in osteoblasts, and the higher RANKL/OPG ratio increases the osteoclastogenesis. (c) CCN3 inhibits osteoblasts differentiation and thus reduces bone formation.

Interestingly, evidence on CCN family proteins in metastatic bone tumors indicates their pivotal role in bone microenvironment (Figure 1). However, there remains a lack of sufficient studies on CCN proteins in prostate and lung cancer metastasis to the bone. Further studies are required to confirm the molecular basis of CCN proteins in metastatic bone tumors. Moreover, the correlation and commonality of CCN proteins in metastatic bone tumors will help elucidate the importance of CCN family proteins in the bone microenvironment.

CTGF, one member of the CCN family associated with tumorigenesis, is a novel therapeutic target for the treatment of pancreatic cancer and is currently being investigated in a Phase I clinical trial. The results showed that twice weekly i.p. administration of FG-3019, a fully human CTGF-specific monoclonal antibody, decreased tumor growth and metastasis and attenuated tumor angiogenesis and cancer cell proliferation [102, 103]. The other CCN proteins may use similar strategies to develop potential therapeutics which target CCN proteins and apply to bone tumor treatment.

## Figures and Tables

**Table 1:** CCN proteins in primary bone cancers.

Cancer	CCN proteins	Expression level	Experimental observation	References
Osteosarcoma	CCN1	Higher	CCN1 associates with poor prognosis, tumour stage, metastasis and mortality	[62]
			CCN1 knockdown inhibits osteosarcoma cell invasion, migration, and lung metastases	[63]
	CCN3	Higher	CCN3 expression level is associated with higher risk to develop lung metastases	[64]
	CCN4	Higher	CCN4 associates with tumor stage and enhances the migration of osteosarcoma cells by increasing MMP-2 and MMP-9 expression	[65]

Ewing's sarcoma	CCN3	Higher	CCN3 is expressed in approximately 30% of Ewing's sarcomas and associated with lower survival rate	[64]
			High expression of CCN3 is detected in recurrences and metastases when compared to the primary tumor.	[66]
		N/A	Forced expression of CCN3 shows decreased cell proliferation while increased migration and invasion	[67]

Chondrosarcoma	CCN1	N/A	CCN1 enhances the migration of chondrosarcoma cells by increasing MMP-13 expression	[68]
	CCN2	N/A	CCN2 increases the migration through upregulating MMP-13 expression	[69]
	CCN3	N/A	CCN3 increases the migration and expression of matrix metalloproteinase MMP-13	[70]
	CCN4	N/A	CCN4 enhances the migration of chondrosarcoma cells by increasing MMP-2 expression	[71]
	CCN6	N/A	CCN6 enhances the migration of chondrosarcoma cells by increasing ICAM-1 expression	[72]

**Table 2:** CCN proteins in metastatic bone cancers.

Cancer	CCN proteins	Expression level	Expreimental observation	References
	CCN1	Higher	CCN1 associates with poor prognosis, nodal involvement, and metastatic disease	[79]
		N/A	Zoledronic acid downregulates CCN1, thus inhibits tumor growth	[80]
		N/A	Anti-CCN1 neutralizing antibody suppresses primary tumor growth and spontaneous lymph node metastasis *in vivo *	[81]
	CCN2	Higher	The expression of CCN2 is higher in breast cancer bone metastases when compared to normal breast tissue	[82]
		Higher	CCN2 is significantly overexpressed in metastatic tumor cells as compared to disseminated tumor cells	[83]
Breast cancer metastasize to bone		N/A	CCN2 is crucial for osteolytic metastasis and is induced by PKA- and PKC-dependent activation of ERK1/2 signaling by PTHrP	[84]
		Higher	CCN2 expression is further increased by the prometastatic cytokine TGF*β*	[85]
	CCN3	Lower	Expression of CCN3 is lower in tumor when compared to normal specimens	[79]
		Higher	CCN3 is highly expressed in the bone metastases when compared with the other metastases (lung, brain, and liver)	[86]
		Higher	CCN3 is highly expressed in bone metastasis samples from breast cancer patients	[87]
	CCN6	Lower	CCN6 expression is inversely correlated with invasive breast carcinomas	[88]

	CCN1	N/A	CCN1 increases tumorigenesis and metastasis of prostate cancer cells	[89]
Prostate cancer metastasize to bone	CCN3	N/A	Knockdown of CCN3 expression decreases cell migration *in vitro* and tumor growth in bone and bone metastasis *in vivo *	[90]
		Higher	CCN3 expression levels are higher in bone metastasis patients and positively correlated with malignancy in human prostate cancer cells	[91]
	CCN4	Higher	Higher expression level of CCN4 has been found in the tissues and sera of prostate cancer patients in early stages	[92]
